# Immune Response, Safety, and Survival and Quality of Life Outcomes for Advanced Colorectal Cancer Patients Treated with Dendritic Cell Vaccine and Cytokine-Induced Killer Cell Therapy

**DOI:** 10.1155/2014/603871

**Published:** 2014-07-17

**Authors:** Hui Zhu, Xuejing Yang, Jiali Li, Yanjie Ren, Tianyu Zhang, Chunze Zhang, Jintai Zhang, Jing Li, Yan Pang

**Affiliations:** ^1^Department of Oncology, Tianjin Union Medicine Centre, 190 Jieyuan Road, Hongqiao District, Tianjin 300121, China; ^2^Shanghai Claison Biotechnology Co. Ltd, Shanghai, China; ^3^Department of Colorectal Surgery, Tianjin Union Medicine Centre, Tianjin 300121, China; ^4^Department of General Surgery, Tianjin Union Medicine Centre, Tianjin 300121, China

## Abstract

*Purpose.* To determine the immune response after dendritic cell (DC) vaccine and cytokine-induced killer cells (CIK) therapy and assess its associated toxicity, survival benefit, and changes in the quality of life (QOL) of advanced colorectal cancer (CRC) patients. *Methods.* We recruited 100 patients with unresectable CRC orrelapsed CRC after surgery who received DC vaccine and CIK cells (group immunotherapy, group I), and, as a control, 251 patients who had similar characteristics and underwent similar treatments, except for this immunotherapy (group nonimmunotherapy, group NI). After a follow-up period of 489.2 ± 160.4 days, overall survival (OS) of the two groups was compared using the Kaplan-Meier method. *Results.* In group I, 62% of patients developed a positive delayed type hypersensitivity response, and most patients showed an improvement in physical strength (75.2%), appetite (74.2%), sleeping (72.1%), and body weight (70.1%). Adverse events were fever (29.5%), insomnia (19.2%), anorexia (9.1%), sore joints (5.4%), and skin rash (1.0%). No toxicity was observed in patients treated with DC vaccine and CIK therapy. OS was significantly longer in group I than in group NI (*P* = 0.043). *Conclusion.* DC vaccine and CIK therapy were safe and could induce an immune response against CRC, thereby improving QOL and prolonging OS.

## 1. Introduction

Colorectal cancer (CRC) is one of the most common cancers worldwide, and more than half of the patients with this malignancy will die from their disease [1–3]. Surgery, chemotherapy, and radiotherapy are the standard treatment modalities for CRC, which have provided significant benefits for patients [4–6]. For early-stage CRC patients, resection followed by adjuvant chemotherapy and radiotherapy is the preferred treatment strategy, resulting in a 5-year survival rate of 70% to 80% [7]. However, early-stage CRC is asymptomatic, and consequently this malignancy is often not diagnosed until it has reached an advanced stage. Furthermore, most patients with early-stage CRC will relapse and eventually develop advanced CRC, which has a poor prognosis, with a 5-year survival rate of 5% or less [7]. In this situation, chemotherapy is regarded as the first-line treatment.

The adverse effects of these routine therapies are a major problem, and severe treatment-related toxicity may result in discontinuation. Further, the poor general health of patients with advanced CRC can prevent the use of standard therapies [8, 9]. In addition to the problem of severe adverse effects, routine therapies often do not lead to complete tumor eradication in advanced CRC patients [10].

New strategies are needed in order to improve the clinical outcomes for these patients. Many patients are in an immunosuppressed state after surgery, radiotherapy, and chemotherapy, and dysfunction of antigen-specific T cells is common. As a result, tumor cells escape from immune surveillance. Recovering anticancer immunity is one possible approach to cancer treatment and is referred to as immunotherapy. Unlike other therapies, immunotherapy may help build an effective and specific immune response, killing tumor cells whilst minimizing toxicity. Dendritic cell (DC) and cytokine-induced killer (CIK) cell-based immunotherapy is one of the most effective means for killing residual cancer cells, which are a leading cause of recurrence and metastasis, and is well tolerated and associated with excellent compliance [11–15].

The purpose of this study was to evaluate the cellular immune response in terms of delayed type hypersensitivity (DTH), improvement in quality of life (QOL), and the safety and survival benefit of DC vaccine and CIK cell therapy in patients with advanced CRC.

## 2. Patients and Methods

### 2.1. Patients

The study was performed at the Department of Oncology, Tianjin Union Medicine Center, Tianjin, China. Patients with advanced CRC were advised to undergo immunotherapy consisting of an autologous DC vaccine and CIK cell treatment (group I) and were asked to provide informed consent. The inclusion criteria were (1) a histological or cytological diagnosis of advanced CRC; (2) hospitalization between February 1, 2012, and August 30, 2012; (3) an unresectable tumor at the first diagnosis and relapsed or metastatic CRC after surgery; (4) adequate kidney, bone marrow, and liver function and normal coagulation.

Patients who met these inclusion criteria but who did not receive immunotherapy consisting of DC vaccine and CIK cell treatment (group NI) were selected as a control group. All patients were followed up until November 14, 2013. Overall survival (OS) was compared between the two groups and the benefit of the DC vaccine and CIK cell-based immunotherapy regimen were evaluated on this basis (Table 1).

### 2.2. Study Design

This was an open-label, single-institution, parallel-group, nonrandomized, retrospective study performed at the Department of Oncology, Tianjin Union Medicine Center, Tianjin China, between February 1, 2012, and August 30, 2012. The study complied with the class III medical techniques described in “Treatment with autologous immune cells (T cells, NK cells)” that was published by the Chinese Ministry of Health. The protocol was approved by the hospital's ethics committee. All patients provided written informed consent before treatment.

### 2.3. Treatment Schedules

For DC vaccine and CIK cell therapy, peripheral blood mononuclear cells (PBMCs) were collected on day 0. Subsequently, 1 × 10^7^ DCs were infused intravenously for the first three weeks and intradermally for the last three weeks from day 8, and 1 × 10^9^ CIK cells were infused intravenously once a day for 4 days from day 11.

### 2.4. Preparation of DCs and CIK Cells

DCs and CIK cells were prepared as described previously [16–19]. The cancer cells were well separated from the cultured colon cancer cell line Sw480. These cells were disrupted by ultrasound and then centrifuged at 600 g for 30 min. The supernatants (tumor lysate) were collected and used for pulsing DCs and testing for DTH. PBMCs were collected by leukapheresis using the Fresenius KABI System (Germany) and subsequently cultured in serum-free medium overnight. Adherent and nonadherent cells were separated, and DC vaccine was prepared by culturing the adherent cells in the presence of granulocyte-macrophage colony-stimulating factor, interleukin-4, tumor lysate, and tumor necrosis factor for 7 days. CIK cells were prepared by culturing the nonadherent cells in the presence of interferon-*γ*, CD3 monoclonal antibody, and interleukin-2 for 10 days.

### 2.5. Criteria for Allowing the Clinical Use of DC Vaccine and CIK Cells

After analysis of the immune phenotype markers HLA2DR, CD80, and CD83 for DCs and CD3, CD8, and CD56 for CIK cells by flow cytometry, the cultured samples were checked for contamination by bacteria and fungi, and endotoxin levels were less than 5 EU/kg. A total of 1 × 10^7^ DCs were drawn into a syringe for intradermal vaccination or were mixed with 100 mL normal saline (NS) for intravenous vaccination, and 1 × 10^9^ CIK cells were mixed with 100 mL NS for intravenous infusion.

### 2.6. DTH

DTH tests were performed 1 week after the last DC vaccination by the intradermal injection of 4 *μ*g tumor lysate. Tests were read 48 h later. According to the diameter of induration, the results were classified as strongly positive (>10 mm), positive (5–10 mm), weakly positive (2–5 mm), and negative (<2 mm) (Table 2).

### 2.7. QOL

QOL was evaluated by a general improvement in physical strength, appetite, sleeping, and body weight using a standardized questionnaire. Changes in QOL were classified as major changes, minor changes, no change, and a worsening of the symptom. Major and minor changes were considered as to be an improvement in the general status (Table 3).

## 3. Safety

Adverse events including fever, insomnia, anorexia, joint soreness, and skin rash were monitored during DC vaccine and CIK cell therapy. Several of these events might occur simultaneously in the same patient (Table 4).

### 3.1. OS

**Figure 1:**
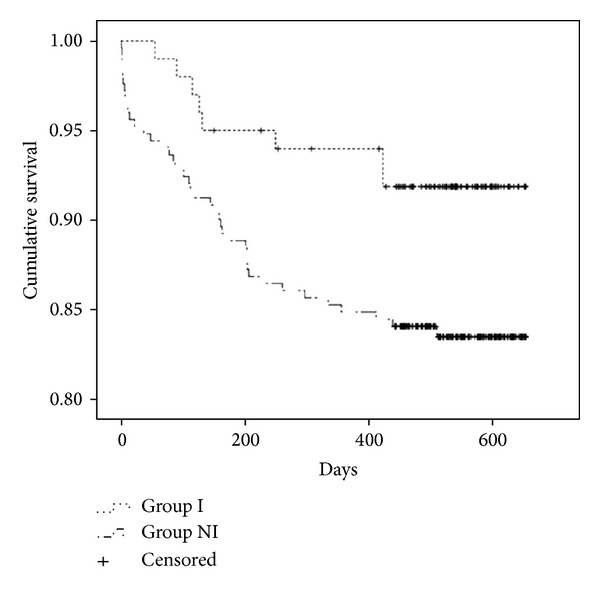
Overall survival curves by the Kaplan-Meier method for patients in group I and group NI. There were a total of 100 patients in group I, 8 of whom died, and a total of 251 patients in group NI, 41 of whom died (*P* = 0.04).

The patients were followed up until November 14, 2013. OS was defined as the survival of patients from the date of enrollment to the date of death due to CRC. Patients who were lost to followup, who died due to an uncertain cause, or whose date of death could not be confirmed were excluded from the OS analysis (Figure 1).

### 3.2. Data Collection and Statistical Analysis

The primary efficacy endpoint for this study was OS. The secondary endpoints were DTH, QOL, and safety. Clinical data were collected from the inpatients electronic medical records of our hospital and reanalyzed and documented for use in this analysis by using Epidata Data Base software (version 3.02, Denmark). Statistical analyses were performed using SPSS (version 19.0) statistical software package, which interfaced with the Epidata Data Base. OS curves were calculated using the Kaplan-Meier method. A *P* value less than 0.05 was considered to be statistically significant.

## 4. Results

### 4.1. Patient Characteristics

A total of 351 CRC patients (229 men and 122 women) were enrolled in this study, with a mean age of 65.9 ± 13.2 years (range, 19–92 years). Patients either received routine treatment alone (251 patients, group NI) or routine treatment plus DC vaccine and CIK cell therapy (100 patients, group I). The characteristics of the patients were well balanced between the two groups, except that more patients in group I underwent surgery (28.0% versus 17.9%, *P* = 0.04). The primary tumor was located in the colon in 108 (30.8%) patients and in the rectum in the other 243 (69.2%) patients. Of the 351 patients, 73 (20.8%) underwent primary tumor resection, 29 (8.3%) received radiotherapy, and 55 (15.7%) received chemotherapy (Table 1).

### 4.2. DTH Skin Test

The DTH skin test was used to assess the immune response to DC vaccine and CIK cell therapy for all patients in group I. Of these 100 patients, 24 patients (24%) had a strongly positive response, 26 patients (26%) had a positive response, and 12 patients (12%) had a weakly positive response. In total, 62% of patients (62 of 100) had a positive immune response and the other 38% of patients (38 of 100) failed to show an immune response (Table 2).

### 4.3. QOL

QOL was recorded as an improvement in the general health of patients, although in group I, this data were only available for 97 out of the 100 patients. Out of these 97 patients, 73 (75.2%) showed a positive improvement in their physical strength, 72 (74.2%) had improved appetite, 70 (72.1%) were able to sleep better, and 68 (70.1%) had an increase in body weight (Table 3).

### 4.4. Adverse Effects

Adverse effects were assessed in 97 out of the 100 patients in group I; no data were available for the remaining 3 patients. Of the 97 patients, 29 (29.5%) developed fever, 11 (19.2%) developed insomnia, 9 (9.1%) developed anorexia, 5 (5.4%) developed joint soreness, and 1 (1.0%) developed skin rash. No toxicity resulted from DC vaccine and CIK cell therapy (Table 4).

### 4.5. OS

The mean follow-up period for patients in this study was 489.2 ± 160.4 days (range, 441–652 days). On the last day of the follow-up period (November 14, 2013), 8 out of the 100 patients in group I had died of CRC and 41 out of the 251 patients in group NI had died of CRC. OS was significantly longer in group I than in group NI (*P* = 0.04; Figure 1).

## 5. Discussion

DCs can prime both a primary and secondary immune response against cancer and function as antigen-presenting cells, and DC vaccine can both initiate and amplify tumor antigen-specific responses through the activation of both T helper cells and cytotoxic T lymphocytes [20–24]. CIK cells are a heterogeneous population with both T cell- and natural killer cell-like characteristics. They have cytotoxic activity and can kill tumor cells both directly and indirectly through stimulation of the host immune system. CIK cells thus have the potential to eradicate residual cancer cells, thereby preventing recurrence after tumor resection. These properties have led to CIK cells being included in immunotherapy strategies against cancer [25–29]. Immunotherapy with DCs and/or CIK cells have been shown to be a potential therapeutic approach against cancers and are now widely used in the clinic for several types of malignancy [30–32].

In this study, we found that 62% of patients (62 of 100) treated with DC vaccine and CIK cells developed a positive cell-mediated cytotoxicity response (Table 2). Subsequently, 75.2%, 74.2%, 72.1%, and 70.1% of 97 patients for whom data were available showed improved physical strength, appetite, sleep, and body weight, respectively (Table 3). In general, the adverse effects resulting from the administration of DC vaccine and CIK cells were mild and self-resolving without special treatments, and no toxicity was observed. Furthermore, our results show that DC vaccine and CIK cell treatment significantly improved the OS of advanced CRC patients compared to those treated with conventional therapies alone.

Our findings suggest that DC vaccine and CIK cell therapy could induce an immune response against CRC, improve QOL, and prolong OS. The therapy was safe with no severe adverse effects and could therefore be tolerated by patients in poor health. It is therefore a potentially beneficial option for patients with advanced CRC.

## 6. Conclusion

Our findings indicate that DC vaccine and CIK cell therapy is an effective and safe treatment for advanced CRC that can potentially overcome the severe adverse effects associated with conventional cytotoxic therapy. This immunotherapy regimen improved both the QOL and OS of these patients and may confer a significant clinical benefit in many cases.

## Figures and Tables

**Table 1:** Patient characteristics.

Characteristics	No. (%)			*P*
	Total	Group I	Group NI	
No.	351	100	251	
Age (years)				
Range	19–92	25–85	19–92	0.47
Mean ± SD	65.9 ± 13.2	62.1 ± 12.4	67.4 ± 13.2	
Gender				
Male	229 (65.2)	71 (71.0)	158 (62.9)	0.15
Female	122 (34.8)	29 (29.0)	93 (37.1)	
Tumor location				
Colon	108 (30.8)	29 (29.0)	79 (31.5)	0.65
Rectum	243 (69.2)	71 (71.0)	172 (68.5)	
Treatment baseline				
Surgery	73 (20.8)	28 (28.0)	45 (17.9)	0.04
Radiotherapy	29 (8.3)	8 (8.0)	21 (8.4)	0.91
Chemotherapy	55 (15.7)	18 (18.0)	37 (14.7)	0.45

**Table 2:** DTH skin test results in the DC vaccine and CIK cell therapy group (*N* = 100).

Results of the DTH skin test	Definition (mm)	No. (%)
Strongly positive	>10	24 (24.0)
Positive	5–10	26 (26.0)
Weakly positive	2–5	12 (12.0)
Negative	<2	38 (38.0)

**Table 3:** QOL in the DC vaccine and CIK cell therapy group (*N* = 100, censored data in 3 cases).

Improvement in general status, number of patients (%)				
	Major (%)	Minor (%)	No change (%)	Worse (%)
Physical strength	50 (51.5)	23 (23.7)	19 (19.6)	5 (5.2)
Appetite	49 (50.5)	23 (23.7)	16 (19.6)	9 (5.2)
Sleeping status	53 (54.6)	17 (17.5)	16 (16.5)	11 (11.4)
Weight	49 (50.5)	19 (19.6)	23 (23.7)	6 (6.2)

**Table 4:** Adverse events resulting from DC vaccine and CIK cell therapy (*N* = 100, censored data in 3 cases).

Event	No. (%)
Fever	29 (29.5)
Insomnia	11 (19.2)
Anorexia	9 (9.1)
Joint soreness	5 (5.4)
Skin rash	1 (1.0)

## References

[1] Boyle P, Leon ME (2002). Epidemiology of colorectal cancer. *The British Medical Bulletin*.

[2] Hawk ET, Limburg PJ, Viner JL (2002). Epidemiology and prevention of colorectal cancer. *Surgical Clinics of North America*.

[3] Shin A, Kim KZ, Jung KW (2012). Increasing trend of colorectal cancer incidence in Korea, 1999–2009. *Cancer Research and Treatment*.

[4] Damin DC, Lazzaron RA (2014). Evolving treatment strategies for colorectal cancer: a critical review of current therapeutic options. *World Journal of Gastroenterology*.

[5] Ke T-W, Liao Y-M, Chiang H-C (2014). Effectiveness of neoadjuvant concurrent chemoradiotherapy versus up-front proctectomy in clinical stage II-III rectal cancer: a population-based study. *Asia-Pacific Journal of Clinical Oncology*.

[6] Laurent M, Paillaud E, Tournigand C (2014). Assessment of solid cancer treatment feasibility in older patients: a prospective cohort study. *Oncologist*.

[7] Ratajczak MZ, Jadczyk T, Schneider G, Kakar SS, Kucia M (2013). Induction of a tumor-metastasis-receptive microenvironment as an unwanted and underestimated side effect of treatment by chemotherapy or radiotherapy. *Journal of Ovarian Research*.

[8] Kurniali PC, Luo LG, Weitberg AB (2010). Role of calcium/ magnesium infusion in oxaliplatin-based chemotherapy for colorectal cancer patients. *Oncology*.

[9] Gallagher DJ, Kemeny N (2010). Metastatic colorectal cancer: from improved survival to potential cure. *Oncology*.

[10] Koido S, Ohkusa T, Homma S (2013). Immunotherapy for colorectal cancer. *World Journal of Gastroenterology*.

[11] Ellebaek E, Andersen MH, Svane IM, Straten PT (2012). Immunotherapy for metastatic colorectal cancer: present status and new options. *Scandinavian Journal of Gastroenterology*.

[12] Xiang B, Snook AE, Magee MS, Waldman SA (2013). Colorectal cancer immunotherapy. *Discovery Medicine*.

[13] Toomey PG, Vohra NA, Ghansah T, Sarnaik AA, Pilon-Thomas SA (2013). Immunotherapy for gastrointestinal malignancies. *Cancer Control*.

[14] Amedei A, Niccolai E, D'Elios MM (2011). T cells and adoptive immunotherapy: recent developments and future prospects in gastrointestinal oncology. *Clinical and Developmental Immunology*.

[15] Cui Y, Yang X, Zhu W, Li J, Wu X, Pang Y (2013). Immune response, clinical outcome and safety of dendritic cell vaccine in combination with cytokine-induced killer cell therapy in cancer patients. *Oncology Letters*.

[16] López MN, Pereda C, Segal G (2009). Prolonged survival of dendritic cell-vaccinated melanoma patients correlates with tumor-specific delayed type IV hypersensitivity response and reduction of tumor growth factor *β*-expressing T cells. *Journal of Clinical Oncology*.

[17] Burch PA, Breen JK, Buckner JC (2000). Priming tissue-specific cellular immunity in a phase I trial of autologous dendritic cells for prostate cancer. *Clinical Cancer Research*.

[18] Liu Y, Zhang W, Zhang B, Yin X, Pang Y (2013). DC vaccine therapy combined concurrently with oral capecitabine in metastatic colorectal cancer patients. *Hepato-Gastroenterology*.

[19] Garg AD, Dudek AM, Agostinis P (2013). utophagy-dependent suppression of cancer immunogenicity and effector mechanisms of innate and adaptive immunity. *Oncoimmunology*.

[20] Galluzzi L, Senovilla L, Vacchelli E (2012). Trial watch: dendritic cell-based interventions for cancer therapy. *Oncoimmunology*.

[21] Ragde H, Cavanagh WA, Tjoa BA (2004). Dendritic cell based vaccines: progress in immunotherapy studies for prostate cancer. *Journal of Urology*.

[22] Bol KF, Tel J, de Vries IJ, Figdor CG (2013). Naturally circulating dendritic cells to vaccinate cancer patients. *Oncoimmunology*.

[23] Tel J, Smits EL, Anguille S, Joshi RN, Figdor CG, de Vries IJM (2012). Human plasmacytoid dendritic cells are equipped with antigen-presenting and tumoricidal capacities. *Blood*.

[24] Wang X, Yu WH, Li J (2014). Can the dual-functional capability of CIK cells be used to improve antitumor effects?. *Cellular Immunology*.

[25] Jäkel CE, Hauser S, Rogenhofer S, Müller SC, Brossart P, Schmidt-Wolf IGH (2012). Clinical studies applying cytokine-induced killer cells for the treatment of renal cell carcinoma. *Clinical and Developmental Immunology*.

[26] Yu J, Ren X, Li H (2011). Synergistic effect of CH-296 and interferon gamma on cytokine-induced killer cells expansion for patients with advanced-stage malignant solid tumors. *Cancer Biotherapy & Radiopharmaceuticals*.

[27] Mesiano G, Todorovic M, Gammaitoni L (2012). Cytokine-induced killer (CIK) cells as feasible and effective adoptive immunotherapy for the treatment of solid tumors. *Expert Opinion on Biological Therapy*.

[28] Shi S, Wang R, Chen Y, Song H, Chen L, Huang G (2013). Combining antiangiogenic therapy with adoptive cell immunotherapy exerts better antitumor effects in non-small cell lung cancer models. *PLoS ONE*.

[29] Hasumi K, Aoki Y, Wantanabe R, Mann DL (2013). Clinical response of advanced cancer patients to cellular immunotherapy and intensity-modulated radiation therapy. *Oncoimmunology*.

[30] Zhong R, Teng J, Han B, Zhong H (2011). Dendritic cells combining with cytokine-induced killer cells synergize chemotherapy in patients with late-stage non-small cell lung cancer. *Cancer Immunology, Immunotherapy*.

[31] Zhan H, Gao X, Pu X (2012). A randomized controlled trial of postoperative tumor lysate-pulsed dendritic cells and cytokine-induced killer cells immunotherapy in patients with localized and locally advanced renal cell carcinoma. *Chinese Medical Journal*.

[32] Yin L, Wang S, Zhang L (2013). Efficacy of dendritic cells/cytokine induced killer cells adoptive immunotherapy combined with chemotherapy in treatment of metastatic colorectal cancer. *Chinese Journal of Cancer Biotherapy*.

